# Experiences of rehabilitation exercise during the transition from hospital to home among older patients following lung cancer surgery: a qualitative study guided by the social ecological model

**DOI:** 10.3389/fonc.2026.1763751

**Published:** 2026-10-20

**Authors:** Luga Dong, Yuanyuan Chen, Yi Shang, Yundong Gao, Jing Fu, Yuanling Gu, Lihong Jin, Xuli Shang

**Affiliations:** 1 Department of Rehabilitation Medicine ,The First Affiliated Hospital of Lishui University, Lishui People's Hospital, Lishui, Zhejiang, China; 2 Department of Nursing, Lishui Hospital of Wenzhou Medical University, Lishui, Zhejiang, China; 3 Department of Nursing and Well-being, School of Nursing and Well-being, Changchun Humanities and Sciences College, Changchun, Jilin, China; 4 Department of Public Health, Lishui Hospital of Wenzhou Medical University, Lishui, Zhejiang, China

**Keywords:** elderly, lung cancer patients, postoperative transitional exercise, qualitative study, social ecological model

## Abstract

**Background:**

Older patients who undergo lung cancer surgery often experience reduced lung function, exercise intolerance, and psychological distress during the transition from hospital to home. Understanding their postoperative rehabilitation exercise experiences is essential for improving rehabilitation exercise experiences and long-term outcomes.

**Objective:**

Guided by the Social Ecological Model (SEM), this study aimed to explore the experiences of rehabilitation exercise among older patients during the transition from hospital to home following lung cancer surgery and to identify multilevel factors influencing their participation.

**Methods:**

A qualitative descriptive design was used. Semi-structured face-to-face interviews were conducted with 13 postoperative older patients who had undergone lung cancer surgery, recruited via purposive sampling. Data analysis was performed using the conventional content analysis method proposed by Graneheim and Lundman.

**Results:**

Three major themes and ten subthemes were identified, corresponding to different levels of the Social Ecological Model: (1) Micro-system level: symptom burden, psychological distress, uncertainty, adaptation, perceived benefits, and habit formation; (2) Meso-system level: family encouragement, companionship, and informational support; (3) Macro-system level: insufficient continuity of medical guidance, limited community rehabilitation resources, and inadequate insurance coverage for rehabilitation.

**Conclusion:**

Rehabilitation exercise during the transition from hospital to home was influenced by individual, family, and healthcare-related factors. The findings may inform individualized exercise guidance, caregiver involvement, and continuity of professional follow-up after discharge.

## 1 Introduction

According to GLOBOCAN 2022, lung cancer was the most frequently diagnosed cancer and the leading cause of cancer death worldwide, with approximately 2.48 million new cases and 1.82 million deaths, accounting for 12.4% of all new cancer cases and 18.7% of cancer deaths, respectively (1). In China, it is estimated that 1,060,600 new cases of lung cancer and 733,300 related deaths occurred in 2022, making lung cancer the leading cancer in both incidence and mortality (2). In the province of Zhejiang, an analysis of cancer registry data from 2021 reveals that lung cancer has emerged as the leading cause of both crude cancer incidence and mortality (3). In the context of population ageing, lung cancer incidence and mortality in China remain higher than the global average, resulting in a substantial disease burden (4). Lung cancer incidence increases with age, and the majority of patients are aged 65 years or older at the time of diagnosis (5, 6). China is experiencing rapid population ageing, which has been accompanied by an increase in the number of older adults diagnosed with lung cancer (5, 6). Surgical resection is the preferred curative treatment for early-stage non-small-cell lung cancer. Despite the enhancement of clinical management strategies for lung cancer, a significant proportion of survivors continue to encounter persistent physical and psychological challenges (7). As has been documented, surgical resection has been found to be associated with subsequent reductions in pulmonary function, exercise capacity, and quality of life (7). However, older adults are particularly prone to postoperative physiological decline, including reduced lung capacity, impaired cardiopulmonary fitness, persistent fatigue, and compromised mobility (8, 9). Exercise has been identified as a non-pharmacological intervention with the potential to enhance exercise capacity and physical activity levels in patients diagnosed with lung cancer (10). Nevertheless, participation in exercise remains challenging for many older patients following lung cancer surgery. As reported in previous studies (11, 12), it has been demonstrated that approximately two-thirds of elderly patients who have undergone lung cancer surgery do not attain the recommended level of physical activity postoperatively. This is due to poor adherence to exercise training and difficulties in sustaining long-term benefits. As indicated by the extant literature, fatigue, pain and respiratory difficulties are prevalent factors that impede the participation of these individuals in physical exercise (13).

In clinical practice, a significant number of patients have been found to have unmet needs for structured rehabilitation programmes and appropriate disease management strategies (14). It is hypothesised that the provision of support to ensure adherence to rehabilitation exercise during the transition from hospital to home may contribute to postoperative recovery (15–17). The exploration of patients’ perspectives on postoperative rehabilitation exercise, encompassing their concerns and support requirements, has the potential to inform the development of sustainable rehabilitation exercise management strategies. Despite the fact that previous studies (18, 19) have examined barriers to rehabilitation exercise participation, the majority have focused on clinician-patient communication, perceptions of disease management, and the social environment. However, there has been a paucity of research on the patients’ own understanding of their condition and the incorporation of rehabilitation exercise into their daily lives. Consequently, an analysis of patients’ experiences in real-world settings, encompassing the challenges they encounter in daily life, the factors that facilitate participation, and the strategies they employ to address these challenges, may facilitate the identification of practical methods to support postoperative rehabilitation.

The Social Ecological Model (SEM) is a revised and standardised term in public health research, serving as a comprehensive framework for understanding health behaviours (20). The emphasis is placed on the interactive effects of multilevel influences, which are comprised of the following: individual physical and psychological factors (micro-system), family and interpersonal networks (meso-system), and broader healthcare systems, community resources, and policy contexts (macro-system). This multilevel theoretical framework has been extensively adopted in the fields of oncology and chronic disease research, as it facilitates the identification of the complex determinants that shape patients’ health-related behaviours (21, 22). Despite the recognised importance of postoperative rehabilitation exercise, there is a paucity of qualitative studies that have explored the experiences of older patients with lung cancer during the transitional recovery period. The extant literature is chiefly concentrated on physiological indicators (8, 9), with comparatively less attention devoted to barriers, facilitators, and adaptation strategies. In order to address this evident knowledge gap, the present study explored the postoperative rehabilitation exercise experiences of older patients following lung cancer surgery through the medium of semi-structured interviews, with the Social Ecological Model acting as the overarching theoretical framework.

## 2 Methods

This qualitative descriptive design employed the conventional content analysis approach to explore and interpret the concept of postoperative rehabilitation exercise experiences among older patients following lung cancer surgery during the transition from hospital to home. In the course of the analysis, manifest and latent concepts were derived from the descriptions provided by the participants. These concepts were then coded, abstracted, and organised into main categories or themes (23).

### 2.1 Study participants

Purposive sampling (23, 24) with maximum variation was utilised to recruit older patients who had undergone surgery for primary lung cancer at Lishui People’s Hospital and were in the transition from hospital to home. In this study, the hospital-to-home transition was operationally defined as the period from hospital discharge to approximately eight weeks after lung cancer surgery, during which patients shifted from structured hospital care to home-based self-management and adapted to postoperative symptoms, rehabilitation exercise, and changes in daily activities. The recruitment process was conducted in 2025, with variation sought in terms of age, sex, educational level, tumour location, and exercise pattern. The inclusion criteria were as follows: The inclusion criteria for this study are as follows (1): pathologically confirmed primary lung cancer (2); having undergone surgery for primary lung cancer (3); age ≥60 years, in accordance with the Law of the People’s Republic of China on the Protection of the Rights and Interests of Older Persons (6) (4); being approximately 8 weeks postoperatively at the time of recruitment and having engaged in or attempted postoperative rehabilitation exercise at home after discharge (5); ability to understand the interview questions and describe personal experiences; and (6) provision of written informed consent. Participants were recruited at approximately 8 weeks after surgery to ensure that they had sufficient experience of home-based rehabilitation while still being able to recall their early experiences of the transition from hospital to home. Exclusion criteria encompassed cognitive impairment that precluded meaningful participation in an interview, concurrent malignancies, or end-stage organ dysfunction.

The sample size was determined by data saturation (25). The collection and analysis of the data were conducted concurrently. Subsequent to each interview, a comparison was made by two researchers between the recently generated codes, sub-themes and themes with the prevailing coding framework. The saturation level was evaluated at both the code and meaning levels. The term ‘code saturation’ was defined as the point at which no new codes, sub-themes or themes were identified. Conversely, the concept of ‘meaning saturation’ was considered to have been reached when the additional interviews provided no new dimensions or interpretations of the existing themes. Following the thirteenth interview, both researchers reviewed the coding framework and concluded that no new codes, themes, dimensions, or interpretations had emerged and that the existing themes had been sufficiently developed. Consequently, the recruitment process was brought to a close, yielding a final sample of 13 participants. Participant characteristics are presented in **Table 1**.

**Table 1:** Participant characteristics (N = 13).

Characteristics	Category	n or (M ± SD; Range)
Age (years)		70.23 ± 5.14
Gender	Male	8
	Female	5
Educational level	Primary	6
	Junior high	3
	Senior high	3
	University	1
Location of lung cancer	Right lung	6
	Left lung	7
Type of lung cancer types	Squamous cell carcinoma	3
	Adenocarcinoma	10
Stage	I	8
	II	2
	III	1
	IV	2
Exercise style	Walk	4
	Dance	1
	Climb stairs	2
	Baduanjin	3
	Aerobics	1
	Respiratory training	2
Frequency of exercise	1 times/D	3
	2 times/D	6
	3 times/D	4
Duration of exercise	≤30 min/times	8
	30–60 min/times	1
	≥60 min/times	4

The present study was approved by the Ethics Committee of Lishui People’s Hospital (Approval No.: 2024-056-01). Prior to participation in the study, written informed consent was obtained from all participants.

### 2.2 Interview guide development

A semi-structured interview guide was developed based on the Social Ecological Model to explore multilevel factors influencing older patients’ experiences of rehabilitation exercise during the transition from hospital to home. The guide comprised questions addressing three core dimensions (1): micro-system factors such as symptom experience, emotions, and exercise confidence (2); meso-system factors including family involvement and social support; and (3) macro-system factors such as medical guidance, community resources, and policy support.

The interview guide was refined through a pilot test involving two postoperative older patients following lung cancer surgery, thereby ensuring its clarity, relevance, and comprehensiveness. Minor refinements were made in light of feedback from pilot participants and consultation with two clinical experts.

### 2.3 Data collection

The data presented herein were collected through one-to-one in-depth interviews. At the commencement of each interview, open-ended questions and non-directive probing strategies were employed to facilitate communication with older patients and establish their trust. Subsequently, the interview guide comprised a series of open-ended inquiries designed to elicit the participants’ accounts of the factors that either facilitated or impeded rehabilitation during the post-discharge transition subsequent to lung cancer surgery. Exploratory and follow-up questions, such as “Could you tell me more about this?” and “Could you explain this more clearly?”, were used. Non-verbal cues, including tone of voice, pauses, facial expressions, and emotional reactions, were systematically documented to deepen contextual comprehension of participants’ responses. At the conclusion of each interview, participants were posed the following question: “Is there any significant information that was not mentioned during the interview and that you would like to add?” Immediately following each interview, detailed field notes were completed in order to record real-time observations and reflective insights. The interviews were conducted in quiet, private rooms within the hospital or the participants’ homes, depending on their preference. No fixed time limit was imposed on the interviews. The duration of the interviews was determined by the necessity to cover the specified interview topics and to provide the participants with sufficient time to articulate their experiences in full. This was further informed by considerations of fatigue and other postoperative symptoms experienced by the participants. The interviews lasted between 30 and 40 minutes and were audio-recorded with the participants’ consent. The process of sampling continued until data saturation (23). It is important to note that all interviews were audio-recorded using an MP3 recorder. The data presented herein were collected through thirteen face-to-face, semi-structured in-depth interviews with thirteen participants.

### 2.4 Data analysis

The interview recordings were transcribed verbatim in Microsoft Word by the interviewer within 24 hours of each interview. In order to verify the accuracy of the transcription, a second researcher compared each transcript line by line with the corresponding audio recording. Following the replaying of the relevant sections of the recording, any discrepancies were rectified. The analysis of the data was conducted using the conventional content analysis approach described by Graneheim and Lundman (24). Each interview transcript was treated as a unit of analysis and read multiple times to gain an overall understanding of the data. In the subsequent stage of the research, the relevant units of meaning pertinent to the study’s objective were identified and condensed, whilst ensuring that their fundamental significance remained unaltered. The condensed meaning units were then assigned initial codes. Two researchers independently coded the interview transcripts and subsequently compared their coding decisions. The group engaged in deliberations concerning various issues, including code labels, meaning-unit boundaries, and theme assignment. These deliberations were conducted until a consensus was achieved. The subsequent development of themes was informed by the identification of both similarities and differences among the codes. As each new interview was subjected to analysis, the emerging themes were reviewed, refined, and merged. This process of amalgamation and refinement ultimately resulted in the identification of the main sub-themes and themes. NVivo 12 Plus was utilised to facilitate the organisation, management and retrieval of the data and codes.

### 2.5 Rigor and trustworthiness

In order to guarantee methodological rigour, Lincoln and Guba’s criteria (26) for trustworthiness were adhered to. The credibility of the research was established through repeated engagement with the data, peer debriefing, and member checking. In the context of member checking, the responsibility for reviewing interview summaries was entrusted to two participants, who were subsequently invited to offer their insights regarding the congruence between the researchers’ interpretations and their personal experiences. Peer debriefing entailed two clinical experts who reviewed the emerging codes and themes and engaged in a discussion about alternative interpretations. The dependability of the study was supported by the documentation of the data collection and analysis procedures, changes to the coding framework, and decisions made during theme development. Confirmability was enhanced through the use of reflexive notes and an audit trail containing coding records, analytic memos, theme revisions, and records of consensus discussions. Transferability was facilitated by employing maximum variation sampling and providing comprehensive descriptions of the participants, the study setting, the data collection procedures, and the research context.

## 3 Results

A total of thirteen participants (eight males and five females) with a mean age of 60–77 years took part in the study. **Table 1** presents a comprehensive overview of the demographic and general information of the participants. Three major themes and ten subthemes were identified, reflecting multilevel influences across the micro-, meso-, and macro-systems that shaped rehabilitation exercise experiences among older patients following lung cancer surgery during the transition from hospital to home.

### 3.1 Theme 1: micro-system level — individual factors

Participants reported a variety of physiological and psychological challenges that directly influenced their rehabilitation exercise engagement. Despite physical limitations, some patients gradually adapted, perceived meaningful benefits, and formed sustainable exercise habits.

#### 3.1.1 Symptom burden

Many patients described dyspnea, incision pain, and decreased physical strength as major barriers that hindered their willingness or ability to exercise. These symptoms often intensified anxiety and reduced confidence in performing rehabilitation activities.

Example quotes:

“Sometimes when I take a deep breath accidentally, I still feel short of breath and very uncomfortable.” (P2)

“Every time I stretch my upper body or do chest expansion exercises, the area around the wound feels like it’s being pulled.” (P10)

#### 3.1.2 Psychological distress and uncertainty

Fear of wound dehiscence, anxiety about incorrect movements, and uncertainty regarding postoperative recovery were frequently reported. These emotional reactions inhibited active participation in rehabilitation exercise routines.

Example quotes:

“I never exercised before … the doctor said to practice daily, but I had no idea where to start.” (P6)

“I always feel that if I exert a little force, the wound will split.” (P4)

#### 3.1.3 Adjustment and adaptation

Some participants gradually learned to adjust their rehabilitation exercise pace and techniques to manage discomfort. These adaptive strategies helped maintain participation despite physical limitations.

Example quote:

“Sometimes I feel a little uncomfortable, but I know it’s normal. I just adjust my movements.” (P12)

#### 3.1.4 Perceived benefits and habit formation

Participants highlighted improvements in breathing, mobility, and overall well-being as important motivators. Over time, several individuals developed fixed rehabilitation exercise routines, maintaining participation during early recovery.

Example quotes:

“After practicing daily, my breathing became smoother.” (P6)

“I practice every morning … I slowly got used to it.” (P7)

### 3.2 Theme 2: meso-system level — family support

Participants described family support as facilitating their exercise participation. Emotional encouragement, shared participation, and informational support collectively boosted patients’ confidence and motivation.

#### 3.2.1 Emotional support

Reassurance and continuous encouragement from family helped alleviate fear and uncertainty during rehabilitation.

Example quote:

“My family kept encouraging me, so I gradually tried.” (P9)

#### 3.2.2 Companionship during rehabilitation exercise

Exercising alongside a spouse reduced feelings of isolation and increased willingness to engage in rehabilitation.

Example quote:

“My spouse said, ‘Let’s practice together.’ I don’t feel so alone anymore.” (P2)

#### 3.2.3 Informational support

Family members often searched for rehabilitation exercise videos, rehabilitation manuals, or professional guidance to help patients perform exercises correctly.

Example quotes:

“My son bought rehabilitation books online and taught me step by step.” (P8)

“They found a video teaching breathing and exercise methods.” (P4)

### 3.3 Theme 3: macro-system level — healthcare, community, and policy factors

Participants reported broader structural factors that limited their rehabilitation process, including insufficient continuity of medical guidance, lack of tailored community programs, and inadequate insurance coverage for postoperative rehabilitation services.

#### 3.3.1 Limited continuity of medical guidance

Although initial in-hospital guidance helped patients build confidence, the lack of personalized follow-up after discharge confused rehabilitation exercise techniques and intensity.

Example quotes:

“The doctor said ‘moderate’, but I still don’t know what my limits are.” (P10)

“Once discharged, no one followed up. I didn’t know how to continue.” (P4)

#### 3.3.2 Insufficient community rehabilitation resources

Community facilities and group activities were often unsuitable for postoperative patients, lacking programs tailored to lung cancer recovery.

Example quotes:

“There are no specialized rehabilitation courses in the community.” (P4)

“The exercise intensity in the community is too high, and I worry it will damage my lungs.” (P11)

#### 3.3.3 Limited insurance and service support

Low reimbursement for rehabilitation therapies created financial burdens and discouraged long-term adherence.

Example quotes:

“Breathing training and physical therapy all cost my own money.” (P11)

“Medical insurance doesn’t cover it after discharge.” (P5)

## 4 Discussion

### 4.1 Summary of main findings

This qualitative study explored postoperative rehabilitation exercise experiences among older patients following lung cancer surgery during the transitional period after discharge using the Social Ecological Model. Three major themes emerged across the micro-, meso-, and macro-systems. At the micro-level, participants confronted symptom burden, psychological distress, and disease-related uncertainty, as well as difficulties in adapting to postoperative rehabilitation exercise. Conversely, a subset of participants exhibited positive adaptation, perceived therapeutic benefits, and the formation of sustainable rehabilitation exercise habits. At the meso-level, family support played a crucial role in enhancing emotional stability, promoting motivation, and improving confidence in performing rehabilitation exercises correctly. At the macro-level, insufficient continuity of medical guidance, inadequate community rehabilitation resources, and limited policy or insurance support posed substantial barriers to long-term rehabilitation exercise experiences.

### 4.2 Comparison with existing literature and current guidelines

Our findings are consistent with previous studies showing that postoperative symptoms, including dyspnea, pain, and fatigue, may reduce older patients’ willingness to participate in rehabilitation exercise. Gu et al. (27) similarly reported that symptom severity was associated with lower adherence and poorer functional recovery. Psychological distress and fear were also identified in this study, particularly uncertainty about rehabilitation exercise techniques and safety. Although current guidelines recommend exercise assessment and individualized exercise management (28), participants reported that such guidance was not provided consistently after discharge. Family involvement was described as supporting rehabilitation confidence and exercise performance. Previous research has also identified caregiver companionship, informational support, and shared participation as facilitators of postoperative rehabilitation among patients with lung cancer (29). However, the current lung cancer rehabilitation guideline explicitly includes caregiver participation only in its recommendation for dyadic yoga and provides limited guidance on caregiver involvement in routine postoperative rehabilitation exercise (28). Our findings suggest that future guideline updates could consider caregiver education and involvement in rehabilitation exercise guidance and emotional and motivational support. At the system level, discontinuity in medical guidance, limited community rehabilitation resources, and financial or insurance constraints were reported as barriers to maintaining rehabilitation, consistent with previous studies (30).The findings, when considered as a whole, indicate that the provision of rehabilitation support during the transition from hospital to home may encompass individualised exercise guidance, early professional follow-up, and caregiver involvement, contingent on patient consent. Caregivers may benefit from information on exercise monitoring, warning signs, and ways to provide emotional and motivational support (31).

### 4.3 Theoretical implications

This study contributes to the advancement of the Social Ecological Model in the context of postoperative rehabilitation among older patients following lung cancer surgery. As demonstrated in the preceding research (32–34), the model has been applied to health promotion and chronic disease management. Nevertheless, there is a paucity of research exploring its utility in transitional postoperative contexts. The identification of influences across the micro-, meso- and macro-systems suggests that multilevel interventions may be useful. Theoretically, the results underscore the dynamic interplay between personal experiences (symptoms, emotions), social relationships (family involvement), and system-level conditions (healthcare and policy environments). These findings suggest that individual, family, and system-level factors should be considered together when examining rehabilitation exercise during the hospital-to-home transition. The framework may guide the development of multilevel support, but the effects of such support require further evaluation.

### 4.4 Strengths and limitations

Strengths of this study include its qualitative descriptive design, structured data analysis process, and strategies to enhance trustworthiness, such as member checking and audit trails. Furthermore, the demographic and clinical diversity of the sample enabled capture of diverse participant perspectives on subjective experiences of older patients following lung cancer surgery. However, several limitations of the present study should be acknowledged. First, the sample was restricted to a single hospital in one geographic region, which may restrict the transferability of the study findings. Second, the reliance on participants’ self-reported experiences renders the data susceptible to recall bias and social desirability bias. Third, although data saturation was achieved in this study, larger-scale, multisite qualitative investigations or mixed-methods research would likely yield more comprehensive and in-depth insights.

## 5 Conclusion

The present study investigated the experiences of elderly patients undergoing rehabilitation exercise during the transition from hospital to home following lung cancer surgery. Participants described factors at three levels as relevant to their rehabilitation exercise: micro-system factors, including symptom burden, psychological responses, adaptation, and perceived benefits; meso-system factors, including emotional, motivational, and informational support from family members; and macro-system factors, including continuity of professional guidance and access to rehabilitation resources. The present study contributes to extant knowledge by providing a context-specific account of rehabilitation exercise during early home-based recovery, by applying an adapted three-level Social Ecological Model framework. The findings may inform rehabilitation support that includes individualised exercise guidance, caregiver education and involvement, and continuity of professional follow-up during this transition. It is recommended that future studies evaluate the feasibility and effects of such multilevel support in different healthcare settings.

## Data Availability

The original contributions presented in the study are included in the article/**Supplementary Material**. Further inquiries can be directed to the corresponding authors.
